# Changes in Attitudes of Life Insurance Companies Towards Patients with Sleep Apnea Syndrome Undergoing Continuous Positive Airway Pressure in Japan

**DOI:** 10.14789/jmj.JMJ22-0026-OA

**Published:** 2022-12-01

**Authors:** DAISAKU SAWADA, KIYOHIDE TOMOOKA, TAKESHI TANIGAWA

**Affiliations:** 1 Department of Public Health, Juntendo University Graduate School of Medicine, Tokyo, Japan; 1 Department of Public Health, Juntendo University Graduate School of Medicine, Tokyo, Japan

**Keywords:** CPAP treatment, acceptance criteria, life insurance company, traffic accident

## Abstract

**Objective:**

Recent studies have revealed that sleep apnea syndrome (SAS) increases the risk of cardiovascular diseases and their risk factors, as well as the risk of traffic accidents. Although SAS screening and early treatment are important, truck drivers may avoid SAS screening to prevent the denial of their application for life insurance due to receiving continuous positive airway pressure (CPAP) treatment. Thus, this study investigated how life insurance companies handle patients on SAS treatment.

**Material and Methods:**

We conducted a questionnaire survey on how they handle life insurance applications of patients with SAS on CPAP treatment for 46, 41, and 42 companies in 2009, 2015, and 2021, respectively, and analyzed the changes in their handling of life insurance applications of patients with SAS.

**Results:**

The results revealed that while about 10 life insurance companies handle the application of life insurance of patients on CPAP treatment in the same way as healthy individuals, many life insurance companies handle them differently. This survey also revealed the differences in handling patients with SAS on CPAP treatment among companies and their policies regarding the reasons.

**Conclusions:**

The survey revealed that there are differences among life insurance companies in handling patients with SAS on CPAP treatment. It is important to provide information about the companies that would not give disadvantages to patients with SAS on CPAP treatment who purchase life insurance. It is also crucial to provide life insurance companies with evidence of reduced risk of traffic accidents in patients with SAS on CPAP treatment.

## Introduction

Obstructive sleep apnea (OSA) is characterized by frequent episodes of upper airway collapse and partial collapse during sleep. It is also associated with congestive heart failure, stroke, atrial fibrillation, ischemic heart disease, and systemic hypertension^1)^. OSA has also been associated with the development of diabetes^2, 3)^. The odds ratio of work accidents was 2.2 (95% confidence interval=1.5-3.1) in workers with OSA^4)^, which is consistent with a 2.5-fold increase in motor vehicle crash risk with OSA estimated by a meta-analysis^5)^.

Continuous positive airway pressure (CPAP) treatment is effective in treating sleep apnea syndrome (SAS). It improves the risk of traffic accidents to the level of healthy individuals, according to a previous report^6)^.

The Ministry of Land, Infrastructure, Transport, and Tourism (MLIT) has established the “Manual for sleep apnea syndrome measures in motor carrier operators - Use of the necessity of SAS measures”^7)^ for occupational drivers and transportation companies. They clarified the points that business operators should pay attention to in each process, starting from a screening test, to a detailed examination of treatment. They strongly recommend SAS screening and adequate treatment for commercial drivers.

Meanwhile, the Japan Trucking Association received a report from one driver on his purchase of group credit life insurance, which is essential for house mortgages, and was rejected because of his CPAP treatment for SAS. Therefore, despite the importance of early detection of SAS through SAS screening and subsequent early treatment to prevent traffic accidents, concerns have been raised that truck drivers would refuse to undergo SAS screening to avoid the refusal of life insurance screening, and that the number of untreated and undiagnosed SAS patients engaging in driving duties would increase. It may not only be a health issue for individual patients, such as an increased risk of cardiovascular diseases, but also pose a danger to the entire society the risk of traffic accidents may increase.

Based on the above background, in this study, we aimed to clarify the actual status of handling of life insurance applications by patients with SAS and changes in the handling of such patients by life insurance companies in Japan. We conducted questionnaire surveys in all life insurance companies in Japan in 2009, 2015, and 2021.

## Material and Methods

The study targets were all life insurance companies in Japan in 2009, 2015, and 2021, and the same questionnaire regarding the handling of life insurance applications by patients with SAS was administered to 46, 41, and 42 companies, respectively.

The questionnaire forms were sent to persons in charge in the corresponding insurance companies, and their responses were regarded as consent for this study. Because this study was a questionnaire survey of life insurance companies and did not collect data from patients or healthy individuals, approval from the institutional review board was waived.

Regarding the criterion for accepting the application of patients with SAS on CPAP treatment, we asked, “Please let us know the criteria for acceptance in your company in the event that a SAS patient on CPAP treatment wishes to purchase life insurance from your company.” If the answer was either “We will accept the application in the same way as healthy individuals since it is in the acceptance criteria” or “We will accept the application in the same way as healthy individuals although it is not in the acceptance criteria,” it was regarded as “Accept in the same way as healthy individuals.” If the answer was either “We will accept the application by changing the premium since it is in the acceptance criteria” or “We will accept the application by changing the premium although it is not in the acceptance criteria,” it was regarded as “Accept by changing the premium.” If the answer was “We will not accept the application,” it was regarded as “Not accept (denial).” Companies that did not return their answer sheets and those that returned answer sheets without responses were regarded as “Not responded.” We also asked about the reasons for each response and the conditions necessary for patients with SAS on adequate CPAP treatment who wish to purchase life insurance to make a contract under the same conditions as healthy individuals.

## Results

Table 1 shows the changes in the acceptance criteria for patients with SAS on CPAP treatment who wished to purchase life insurance. The number of companies that answered “We will accept the application in the same way as healthy individuals” was 10 (22%) in the survey in 2009, 8 (20%) in 2015, and 12 (29%) in 2021, showing that there are approximately 10 companies with slight increases or decreases. The number of companies that answered “We will accept the application by changing the premium” was 7 (15%) in the survey in 2009, 4 (10%) in 2015, and 6 (14%) in 2021. The number of companies that answered “Not accept” was 1 (2%) in the survey in 2009, 1 (2%) in 2015, and 0 (0%) in 2021.

**Table 1:** Changes in the acceptance criteria for patients with SAS for CPAP^a)^ treatment who wish to purchase life insurance

Survey year (number of companies)	2009 (46)	2015 (41)	2021 (42)
Acceptance criteria Accepted in the same way as healthy individuals	10（22%）	8（20%）	12（29%）
Accepted by changing the premium	7（15%）	4（10%）	6（14%）
Not accepted	1（2%）	1（2%）	0（0%）
Other	0（0%）	2（5%）	0（0%）
No response to this survey	28（61%）	26（63%）	24（60%）

^a)^CPAP: continuous positive airway pressure *Percentages are rounded to the nearest whole number.

When we asked why they did not accept these applications under the same conditions as healthy individuals, 8 companies responded to the survey in 2009, 5 in 2015, and 4 in 2021. These companies gave various reasons, such as “CPAP is a treatment, and it is not possible to accept the application under the same conditions as healthy individuals” and “It is difficult to individually grasp the compliance with treatment,” indicating that the reasons for not accepting the life insurance application vary by company (Table 2).

**Table 2:** Reasons for not accepting the application of patients with SAS on CPAP^a)^ treatment, etc. in the same way as healthy individuals

Company	2009	2015	2021
#1	It is difficult to check individual compliance with treatment such as CPAP.	We decide that there is a risk in the first 6 months. We accept patients for CPAP treatment after 7 months of treatment for insurance of death, severe disability, etc., in the same way as healthy individuals.	The decision of acceptance may be strict, as there are patients who discontinue the use of CPAP at the start of treatment because it does not suit them or those who cannot use CPAP appropriately.
#2	No data on SAS are accumulated in the company.		
#3	We sometimes accept the application on special conditions or sometimes deny the application even if customers have a disease under treatment or have a medical history that is considered to have a relatively good clinical prognosis.		
#4	We do not accept any application of people under treatment not limited to SAS.	The start of CPAP treatment does not mean the confirmation of the final treatment effect. So, we cannot accept the application.	We limit the amount of compensation for a certain period after acceptance.
#5	We make a decision for acceptance individually according to symptoms, etc.,		
#6	Underlying diseases such as obesity and hypertension are likely to be present. In addition, there is an issue of continuation of (compliance with) CPAP treatment.		
#7	It is difficult to predict the status of subsequent long-term treatment and the status of continuation at the time of accepting the application.		
#8	Since the treatment is conducted in a self-management manner, it is not possible to determine whether or not the treatment is performed accurately or whether or not the effect is increased.		
#9		CPAP is a symptomatic treatment and requires a device to be worn at bedtime every night (= needs efforts to encourage the insured to continue treatment). Therefore, we consider it necessary to make certain careful decisions on the acceptance of the application for life insurance which is characterized by long-term insurance coverage.	
#10		The criteria have been established based on past statistics, accident occurrence data, etc.	
#11		Since CPAP is a treatment, we will not accept it as a standard case. However, since the risk of onset is considered to be low, it is handled with extra premium.	It is handled as a disease without distinguishing it from other diseases.
#12			It is assessed considering treatment compliance, risks, etc.

^a)^CPAP: continuous positive airway pressure

Similarly, it was revealed that each company holds a different view on the conditions necessary to accept the application of patients with SAS on adequate CPAP treatment who wish to purchase life insurance under the same conditions as healthy individuals (Table 3).

**Table 3:** Conditions necessary for patients with SAS adequately treated with CPAP^a)^, etc. who wish to purchase life insurance to make a contract under the same conditions as healthy individuals

Company	2009	2015	2021
#1	It is necessary for a patient to present a medical certificate from a doctor who can confirm the course and effect of treatment. Anyway, we will accept the application with a special insurance premium.	We decide that there is a risk in the first 6 months. We accept the application of patients on CPAP treatment after 7 months of treatment for insurance of death, severe disability, etc., in the same way as healthy individuals.	After a certain period of time after the start of CPAP treatment.
#2	Since no medical data for insurance on SAS has been accumulated in the company, we cannot accept the application of patients with SAS in the same way as healthy individuals.		
#3	If a patient is under treatment for SAS, we accept the application with a special condition based on our criteria.		
#4	We do not accept any application of people under treatment not limited to SAS.	Only when a patient finishes treatment, including surgery (complete cure), and it is confirmed that there is no complication, such as hypertension and diabetes mellitus, we will consider the acceptance of the application.	We accept the application by limiting the amount of compensation for a certain period after acceptance.
#5	^b)^We make a decision for acceptance individually according to symptoms, etc.		
#6	It is necessary for a patient to present medical documents that show the absence of underlying diseases such as obesity and hypertension and can resolve the issue of continuation of (compliance with) CPAP treatment.		
#7	In the event of cure by weight loss, surgery, etc.	^b)^Certificate of cure	^b)^We mostly accept the application without conditions.
#8		Since CPAP is a treatment, we will not accept it as a standard case. However, since the risk of onset of diseases is considered to be low, it is handled with extra premium.	A patient should be younger than 50 years and using CPAP.
#9			We do not handle the case under the same conditions as healthy individuals.
#10	^b)^A contract can be made if at least 3 months have passed since the start of treatment and hospitalization or surgery has not been recommended.	^b)^Abnormalities, in addition to SAS, may affect the acceptance or denial.	
#11	^b)^We do not handle any insurance products that require medical underwriting.		^b)^If there is no notification of complications such as hypertension or severe obesity, we will accept the application as usual. However, since SAS has a base score, it is more likely that the application of the patient cannot be accepted compared with healthy individuals. For example, if the patient shows hypertension that can be barely accepted in healthy individuals, SAS patients are more likely to be denied due to the total score of blood pressure and SAS.
#12	^b)^If the patient is treated with CPAP, etc., and is found to be in good condition, the contract can be made under the same conditions as healthy individuals.		
#13	If the patient is not receiving inpatient treatment, we can accept the application.		
#14	^b)^Since the insurance products currently available are a selective type, there are no conditions.	^b)^No particular conditions are set for SAS and CPAP.	^b)^We accept the application of the patients in the same way as healthy individuals.
#15		Abnormalities, in addition to SAS, may affect the acceptance or denial.	We accept the application of the patients in the same way as healthy individuals.
#16		Since our assessment criteria are confidential, we cannot answer the question.	
#17		If it is confirmed by the attending doctor's medical certificate that CPAP treatment has been continued as instructed and treatment is effective.	
#18		^b)^We decide based on the duration from the start of wearing CPAP and the frequency of its use.	
#19		We decide based on the notification of health status from the applicant.	
#20		^b)^At least 3 months have passed since the start of treatment. There is no abnormality in the cardiovascular system, etc. We can accept the application during CPAP treatment.	^b)^We can accept the application during CPAP treatment, but it is difficult to accept if there is a complication.
#21			^b)^No interference with daily activities and no complications.
#22			If a patient is under treatment, it is not possible for the patient to purchase the insurance under the same conditions (unconditional) as healthy individuals. However, the patient may be able to purchase the insurance under nearly the same conditions as healthy individuals depending on the mitigation measures for assessment.
#23			^b)^Blood pressure, glucose metabolism, HDL/LDL cholesterol, BMI, etc., are within the specified range.
#24			^b)^If there is no history of hospitalization for 2 weeks or more and at least 1 year has passed after the start of treatment, we accept the application under the same conditions as healthy individuals.
#25			^b)^There are conditions: the patient is stable with treatment such as CPAP, the degree of obesity is within our acceptance range, there is no causative or underlying disease, and the patient did not develop the disease at a young age. We make a comprehensive decision, including age, physique, treatment method, and underlying disease.

^a)^CPAP: continuous positive airway pressure ^b)^The company that answered that they would accept the insurance application of SAS patients treated with CPAP under the same conditions as healthy individuals.

## Discussion

Approximately 10 life insurance companies answered that they would accept the application of patients with SAS on CPAP treatment in the same way as healthy individuals, which is about 20% to 25% of all insurance companies. Additionally, as shown in Table 3, it was found that various conditions must be met, even at insurance companies that answered that they would accept the application in the same way as healthy individuals.

Accumulating evidence suggests that adequate treatment of OSA with CPAP reduces health and safety risks. Meta-analysis data demonstrated that CPAP treatment significantly reduced OSA severity, sleepiness, blood pressure, and motor vehicle accidents in patients with OSA^8)^. OSA is also linked to adverse effects on employees’ healthcare costs and workplace productivity^9)^. Burks et al. reported that mandatory OSA screening and CPAP treatment programs lead to substantial savings in medical costs^10)^.

Burks et al. published the first large-scale program to screen, diagnose, and monitor OSA treatment adherence among truck drivers. Drivers with OSA who did not adhere to CPAP had a five-fold greater rate of crashes compared to the control group. They also reported that drivers with OSA who were fully compliant with CPAP treatment had a crash rate that was similar to that of the control group^11)^. These data show that OSA is an underdiagnosed disease with preventable adverse effects not only on health but also on work and traffic accidents. Therefore, workplace screening for OSA, especially in safety-sensitive occupations, should be encouraged^12-14)^.

However, the results of this survey suggest that the overall recognition of the effects of CPAP treatment on SAS patients differs among insurance companies. Companies, such as #1 in Table 2, changed their comments in a favorable direction over the three surveys. This is probably because it updated its acceptance criterion based on the latest knowledge and the social environment.

Meanwhile, the criterion for accepting the application varies among life insurance companies. By informing drivers about life insurance companies that accept the application of patients on CPAP treatment in the same way as healthy individuals, it may be possible to prevent drivers from refusing SAS screening because they cannot purchase life insurance.

The results of this survey revealed that some health insurance companies still do not accept the life insurance application of patients with SAS on CPAP treatment in the same way as healthy individuals, and this situation has not changed over the last 10 years. The handling of these patients by life insurance companies affects SAS screening and is an important factor for the continuation of treatment. Therefore, it is necessary to take measures to prevent patients with SAS from being disadvantaged in purchasing life insurance, for example, by disclosing the names of the companies that handle such patients in the same way as healthy individuals. Since each life insurance company has different criteria for the acceptance of the life insurance application of patients with SAS, it is important to promote efforts, such as presenting evidence on the effect of CPAP treatment in reducing the incidence of accidents and disease risks to each insurance company.

In this article, we discuss the problem of truck drivers being treated unfavorably in life insurance coverage due to their CPAP treatment. However, this issue is not limited to truck drivers, but is also important for all workers and retirees who work in various occupations.

## Funding

A part of this study was funded by the International Association of Traffic and Safety Sciences.

## Author contributions

This manuscript was drafted by DS and TT. The questionnaire was compiled by DS and TT. Data interpretation was performed by DS, KT, and TT. All the authors have read and approved the final manuscript.

## Conflicts of interest statement

DS serves as the president of the Institute for Sleep Health, a non-profit organization, without compensation.
